# Phenotypic disruption of cuticular hydrocarbon production in hybrids between sympatric species of Hawaiian picture-wing *Drosophila*

**DOI:** 10.1038/s41598-022-08635-w

**Published:** 2022-03-22

**Authors:** Thomas J. Fezza, Matthew S. Siderhurst, Eric B. Jang, Elizabeth A. Stacy, Donald K. Price

**Affiliations:** 1 Tropical Conservation Biology and Environmental Sciences, University of Hawaii at Hilo, 200 West Kawili St., Hilo, HI 96720 USA; 2 Department of Chemistry, Eastern Mennonite University, 1200 Park Rd, Harrisonburg, VA 22802 USA; 3 Tropical Crop and Commodity Protection Research, D.K.I, U.S. Pacific Basin Agricultural Research Center, Agricultural Research Service, United States Department of Agriculture, 64 Nowelo Street, Hilo, HI 96720 USA; 4 Present Address: School of Life Sciences, University of Nevada, Las Vegas, USA

**Keywords:** Chemical biology, Evolution

## Abstract

Interspecies hybrids can express phenotypic traits far outside the range of parental species. The atypical traits of hybrids provide insight into differences in the factors that regulate the expression of these traits in the parental species. In some cases, the unusual phenotypic traits of hybrids can lead to phenotypic dysfunction with hybrids experiencing reduced survival or reproduction. Cuticular hydrocarbons (CHCs) in insects are important phenotypic traits that serve several functions, including desiccation resistance and pheromones for mating. We used gas chromatography mass spectrometry to investigate the differences in CHC production between two closely related sympatric Hawaiian picture-wing *Drosophila* species, *Drosophila heteroneura* and *D. silvestris*, and their F1 and backcross hybrid offspring. CHC profiles differed between males of the two species, with substantial sexual dimorphism in *D. silvestris* but limited sexual dimorphism in *D. heteroneura*. Surprisingly, F1 hybrids did not produce three CHCs, and the abundances of several other CHCs occurred outside the ranges present in the two parental species. Backcross hybrids produced all CHCs with greater variation than observed in F1 or parental species. Overall, these results suggest that the production of CHCs was disrupted in F1 and backcross hybrids, which may have important consequences for their survival or reproduction.

## Introduction

Interspecies hybrids that express phenotypic traits far outside the range present in the parental species can provide insights into the factors that regulate the expression of these traits^1^. These unusual phenotypic traits in hybrids can also reflect a type of phenotypic dysfunction in which hybrid individuals experience reduced survival or reproduction^2,3^. Several types of gene interactions may be involved in hybrid disruption, such as *cis*–*trans* regulation or post-transcriptional processes, including mRNA splicing and processing^4,5^. Further, translational alterations resulting in changed amino acids may result in proteins incapable of interacting, thus producing less-fit hybrid phenotypes^6^.

Cuticular hydrocarbons (CHCs) are abundant components of insect cuticles^7,8^ that are produced through complex biochemical processes^9,10^ and involve the interaction of genes on different chromosomes^11^. CHCs display a wide range of distinct compounds that vary across insect taxa and occur as a complex mixture of hydrophobic linear, branched, saturated, and unsaturated compounds^11–13^. CHCs are known to act as pheromones and influence a wide variety of behaviors, including courtship, mate discrimination, learning, aggregation, and dominance^5,8,14^, and they have a strong influence on individual fitness, helping insects to resist starvation^15^, tolerate extreme environments^16^, and prevent desiccation^17–19^. In *D. melanogaster*, the alteration or disruption of genes involved in CHC biosynthesis can result in the complete absence^20^ or over-production^21^ of CHCs, changes that have the potential to negatively impact mating, copulation behavior, and survivability^10,22^.

The Hawaiian picture-wing *Drosophila* are a species-rich radiation that vary in CHCs with some species subgroups displaying more linear alkanes and unsaturated hydrocarbons and other subgroups possessing more monomethylalkanes and dimethylalkanes^13^*.* In the well-studied planitibia subgroup, nearly all species display the same main hydrocarbons with subtle but significant quantitative differences among species in the abundances of specific compounds and limited sexual dimorphism. A single exception, *D. silvestris*, shows strong sexual dimorphism in CHC composition as well as differences in the abundances of compounds in males (e.g. greater 2MeC30 and less 2MeC26) compared to the other species in the subgroup^13^.

*Drosophila heteroneura* and *D. silvestris* are sister species within the planitibia subgroup of the Hawaiian picture-wings^23^ that occupy the same larval host plant (*Clermontia* spp) and have coexisted at several locations on Hawaii Island^24^. Despite their shared ecology and plant host, they are morphologically and behaviorally distinct^25^. Divergence in CHC abundances between *D. silvestris* and *D. heteroneura*—apparently due to evolution of *D. silvestris* males (see above)—occurred recently (i.e., < 1 million years ago)^23^ as the two species became established on Hawaii Island. These two species are known to hybridize in nature, as both F1 and backcross individuals have been collected in the wild^24^. Under laboratory conditions these two species readily hybridize to yield fertile F1 progeny that differs from both species with respect to behavioral and morphological characters^26,27^. F1 hybrid females and males can be mated with each parental species to create backcross individuals that exhibit wide genetic and phenotypic variation due to recombination of the genes from the two parental species^28^. Recent genome comparisons of *D. heteroneura* and *D. silvestris* indicate that there was gene flow between these two species after their arrival on Hawaii Island from an older island^29^, and these species show significant sequence divergence for olfactory and gustatory genes that may be important in chemical communication^30^.

The ability to hybridize species in the laboratory allows the controlled examination of phenotypes in parental, F1 and backcross individuals to better understand the genetic basis of species differences, including differences in the regulation of gene expression. We examined the CHCs in laboratory populations of *D. heteroneura* and *D. silvestris* and their F1 and backcross hybrids. The CHC profiles of F1 and backcross hybrids displayed intermediate abundances of some CHCs and unusual amounts of other CHCs. Notably, a third group of CHCs was completely absence in F1 individuals. The disrupted production of CHCs in hybrids suggests that there are important differences between *D. heteroneura* and *D. silvestris* in the regulation of CHC production that have evolved since these species were founded on Hawaii Island. The importance of these differences for ecological adaptation or reproduction in *D. silvestris* and *D. heteroneura* remains to be determined.

## Materials and methods

### Hawaiian* Drosophila* population rearing

The populations of *D. silvestris* and *D. heteroneura* used in this study were initiated with individuals collected in the wild from the South Kona Forest Reserve, Kukuiopae (e 1: GPS coordinates 19.2972818613052, − 155.8117108345032) on the 16–17th of December 2012 and 29th of December 2009, respectively. Flies were attracted to baits comprising a fermented banana-yeast medium and fermented-mushroom spray spread on sponges and hung one to two meters from the ground near patches of *Cheirodendron trigynum* and *Clermontia* sp. The flies were captured using an aspirator and were immediately transferred to sugar-agar vials. The vials were transported to the University of Hawai’i at Hilo where individuals were identified to species and placed in one-gallon breeding jars. Populations of both species were maintained in an environmentally controlled room, following Hawaiian *Drosophila*-specific rearing procedures described in Price and Boake^27^. F1 hybrids were produced by placing one mature *D. silvestris* or *D. heteroneura* male with one or two *D. silvestris* or *D. heteroneura* females. For each of the two cross types, 50 groups of males and females were founded, with breeding individuals being replaced as they died. Breeding individuals were housed in a mating vial with adult food and a tissue soaked in *Clermontia* spp. leaf tea^27^. Adults were transferred to new vials every 4 days, and old mating vials were placed in larvae-rearing trays. After four weeks, larvae vials were placed in emergence jars, and emerged individuals were aspirated into jars weekly according to their respective genotype and sex. The production of backcross individuals was achieved in the same manner by mating F1 females from each parental cross (*D. silvestris* females x *D. heteroneura* males and *D. heteroneura* females by *D. silvestris* males) to mature males of each parental species (*D. heteroneura* and *D. silvestris*). The two types of backcross males were BC—S, males produced by mating F1 females with *D. silvestris* males; and BC—H, males produced by mating F1 females with *D. heteroneura* males: 20–30 pairs were used for the production of each backcross type.

### Chemical analysis of cuticular hydrocarbons

Cuticular hydrocarbon extractions were obtained by placing individual flies in 4-ml vials which were held at − 80 °C for 10 min. After euthanization, 1 mL of hexane was added to each vial. Vials were then gently agitated for 10 min. The solvent from each sample was then transferred to a new clean 2-mL screw-top vial, and the volume was reduced to 30 uL under a stream of nitrogen gas. Extracts were stored at − 80 °C until used for analysis. All flies used for CHC analysis were 28–30-day-old virgin males and females. In the analysis of F1 hybrids and parental species, 320 ng of eicosane, as an internal standard, was also added to each vial to obtain absolute abundances.

Two gas chromatograph (GC) instruments were used to analyze CHC profiles. GC–MS analysis to identify CHCs was performed on an Agilent (Palo Alto, CA, USA) 6890 N GC interfaced with a Hewlett-Packard 5973 Mass Selective Detector. The GC was equipped with an HP-5MS column (30 m × 0.25 mm ID 0.25-μm film thickness), which was temperature-programmed from 180 to 320 °C at 3 °C min^−1^ following a 1-min delay. The injector temperature was 250 °C with the MS transfer line at 280 °C, and helium was the carrier gas (1.1 ml min^−1^). Detected CHCs were identified based on analyses of their mass spectra, retention indices, and comparison with the NIST08 mass spectral database and literature chromatographic data (Alves et al. 2010).

Quantification of CHCs in *D. heteroneura*, *D. silvestris*, F1 hybrid, and backcross individuals was done using an Agilent 6890 GC equipped with a flame-ionization detector (FID) and an HP-5 column (30 m × 0.25 mm ID 0.25-μm film thickness), with helium as the carrier gas (2.3 ml min^−1^). The injector, in splitless mode, and FID were held at 250 °C and 275 °C, respectively. The oven temperature program ran from 180 to 320 °C at 3 °C min^−1^ following a 1-min delay. Peak areas of major CHCs in each fly were quantified using ChemStation software (Agilent Technologies, Santa Clara, California), and individual compounds were normalized to the standard.

### Statistical analysis

The differences in CHC profiles among parental species, F1 hybrids, and backcross individuals were analyzed using T-tests, ANOVAs, principal components analyses (PCA), and logistic regression analysis using R × 64 3.1.1 and Minitab version 16. Tukey’s multiple-comparison tests were conducted to determine the groups that were significantly different following ANOVA. PCA were conducted to account for the underlying correlation structure among the compounds.

## Results

The nine major CHCs detected were 2-methylhexacosane (2MeC26), 2-methyloctacosane (2MeC28), 2-methyltriacontane (2MeC30), 11 + 13-dimethylhentriacontane (11 + 13MeC31), 11,15-dimethylhentriacontane (11,15diMeC31), 2-methyldotriacontane (2MeC32), 11 + 13-dimethyltritriacontane (11 + 13MeC33), 11,15-dimethyltritriacontane (11,15diMeC33) and 11,15-dimethylpentatriacontane (11,15diMeC35). The pairs of compounds 11MeC31 and 13MeC31, along with 11MeC33 and 13MeC33, are known to coelute and have nearly identical mass spectra making absolute structural identification difficult. Therefore, the abbreviations 11 + 13MeC31 were used to indicate ambiguous identification of these peaks.

### Parental and F1 hybrid analyses

**Figure 1:**
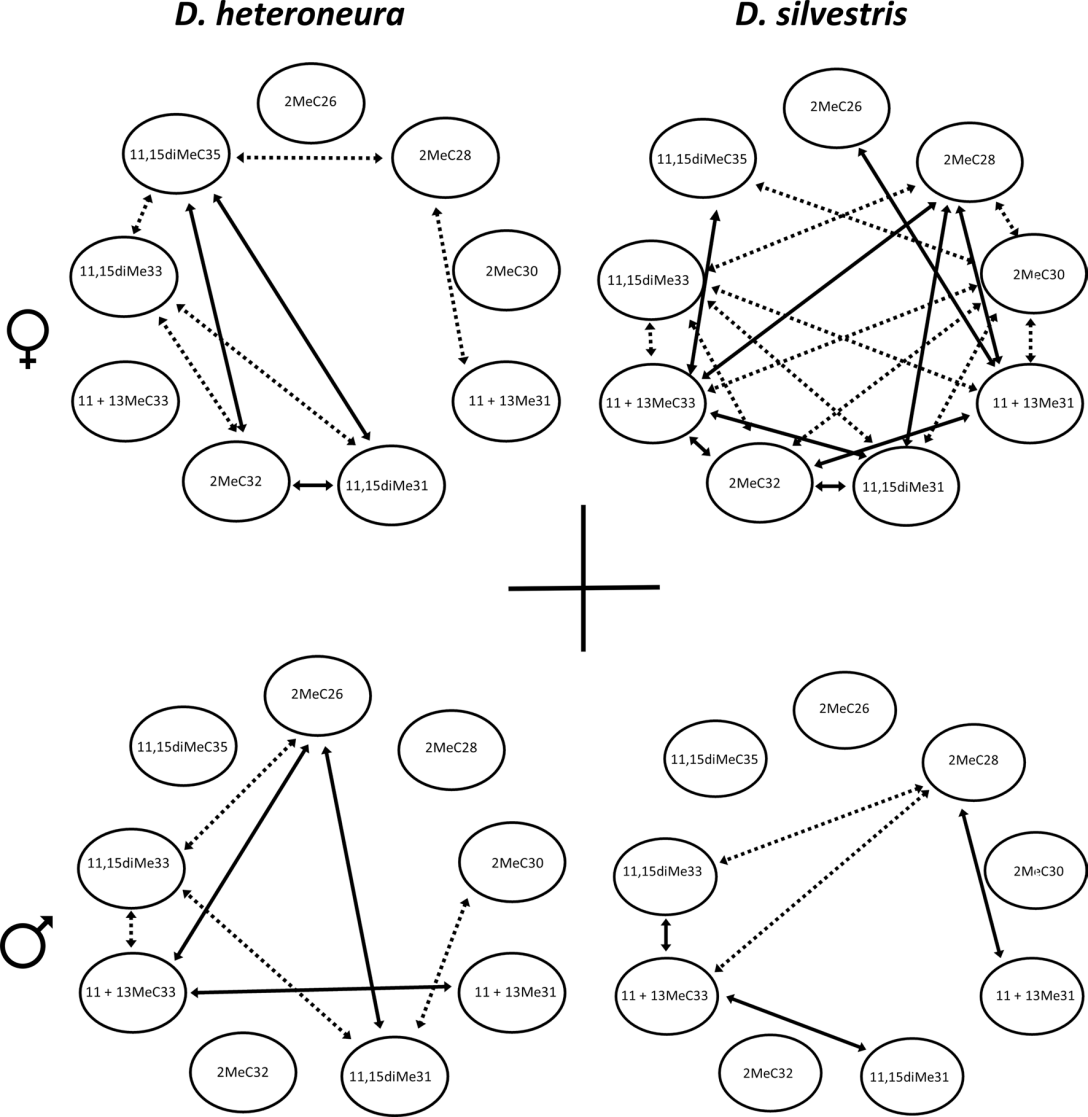
Correlation graph showing the strongest correlations among the nine CHCs in the parental species. *D. heteroneura* females (n = 10) in the upper left, *D. silvestris* females (n = 10) in the upper right, *D. heteroneura* males (n = 10) in the lower left and *D. silvestris* males (n = 10) in the lower right panel. The solid lines indicate the strongly positive correlations (r > 0.6, P < 0.05), and the dashed lines indicate the strongly negative correlations (r < − 0.6, P < 0.05), between compounds. Graph drawn in MSWord from correlations between CHCs in females and males of both species presented in Tables S1 and S2.

The correlation structure of compound abundances differed between both species and sexes (Tables S1 and S2). Viewing just the strongest positive and negative pairwise correlations (i.e. r >|0.6|) between compounds revealed highly constrasting patterns among the four groups (Fig. 1), suggesting variation in the regulation of compounds production between sexes and between species.

Surprisingly, three of the nine compounds detected in the parental species were absent in both F1 females and males: 11 + 13MeC31, 11,15diMeC31, and 2MeC32 (Table S3). The mean relative percent abundances of each of the six CHCs detected in F1 hybrids and each species are reported in Table 1. The relative abundances of the six compounds in *D. heteroneura, D. silvestris* and F1 hybrid individuals were interdependent with some compounds highly significantly correlated (Table S4). In addition, the mean nanogram quantities for all nine CHCs, using the internal chemical standard, showed that the F1 individuals have reduced overall CHC production compared to *D. heteroneura* and *D. silvestris* (Table S3). *D. heteroneura* females also had a slightly lower total CHC production compared to the other parental species individuals.

**Table 1:** Analysis of Variance (ANOVA) of six major CHCs of male and female *D. heteroneura*, *D. silvestris*, and F1 hybrids (n = 30 total individuals, n = 5 for each genotype).

Genotypes	Compounds					
	2MeC26	2MeC28	2MeC30	11 + 13MeC33	11,15diMeC33	11,15diMeC35
*D. heteroneura* Male	9.98 (0.74)^A^	26.33 (0.74)^B^	11.74 (1.18)^D^	4.86 (0.78)^C^	39.04 (1.99)^B^	8.04 (0.89)^A^
*D. silvestris* Male	1.83 (0.62)^C^	34.08 (0.59)^A^	39.84 (0.75)^A^	2.77 (0.53)^D^	17.96 (2.01)^E^	3.53 (0.47)^B^
F1 Male	10.97 (0.49)^A^	33.99 (1.15)^A^	21.59 (1.94)^C^	7.21 (0.92)^B^	22.32 (1.53)^D^	4.04 (1.26)^B^
*D. heteroneura* Female	9.46 (1.30)^A^	25.93(2.07)^B^	8.30 (1.05)^E^	6.27 (0.80)^BC^	42.42 (1.00)^A^	7.64 (1.36)^A^
*D. silvestris* Female	2.67 (0.63)^BC^	20.25 (0.59)^C^	23.63 (2.38)^C^	6.50 (1.58)^BC^	39.47 (1.72)^AB^	7.48 (1.99)^A^
F1 Female	3.75 (1.30)^B^	9.77 (0.52)^D^	33.64 (1.37)^B^	17.41 (0.89)^A^	30.95 (1.60)^C^	4.48 (1.16)^B^
	F = 102.13	F = 299.78	F = 309.98	F = 138.16	F = 180.18	F = 13.07
	df = 5, 24	df = 5, 24	df = 5, 24	df = 5,24	df = 5,24	df = 5,24
	P < 0.001	P < 0.001	P < 0.001	P < 0.001	P < 0.001	P < 0.001

The mean percentage of each compound is reported for each genotype with the standard deviation in parentheses. For each compound, means that do not share a letter are significantly different following a Tukey’s multiple-comparison test(P < 0.05). Compounds 11 + 13MeC31, 11,15diMeC31, and 11,15diMeC31 were not detected in F1 hybrids.

For the six compounds observed in F1 individuals, the PCA of *D. heteroneura*, *D silvestris,* and their F1 hybrid females and males resulted in the first principal component (PC1) explaining 47.2% of the overall variation (Table S5). Three compounds showed positive loadings (2MeC26, 11,15diMe33, and 11,15diMeC35) and three compounds showed negative loadings (2MeC328, 2MeC30 and 11 + 13MeC33). PC2 explained 34.5% of the overall variation and had negative loadings for 2MeC26 and 2MeC28 and positive loadings for the other four compounds. PC3 explained 13.9% of the overall variation with a negative loading of two components (2MeC26 and 11 + 13MeC33) and positive loadings for the other components (Table S5).

**Figure 2:**
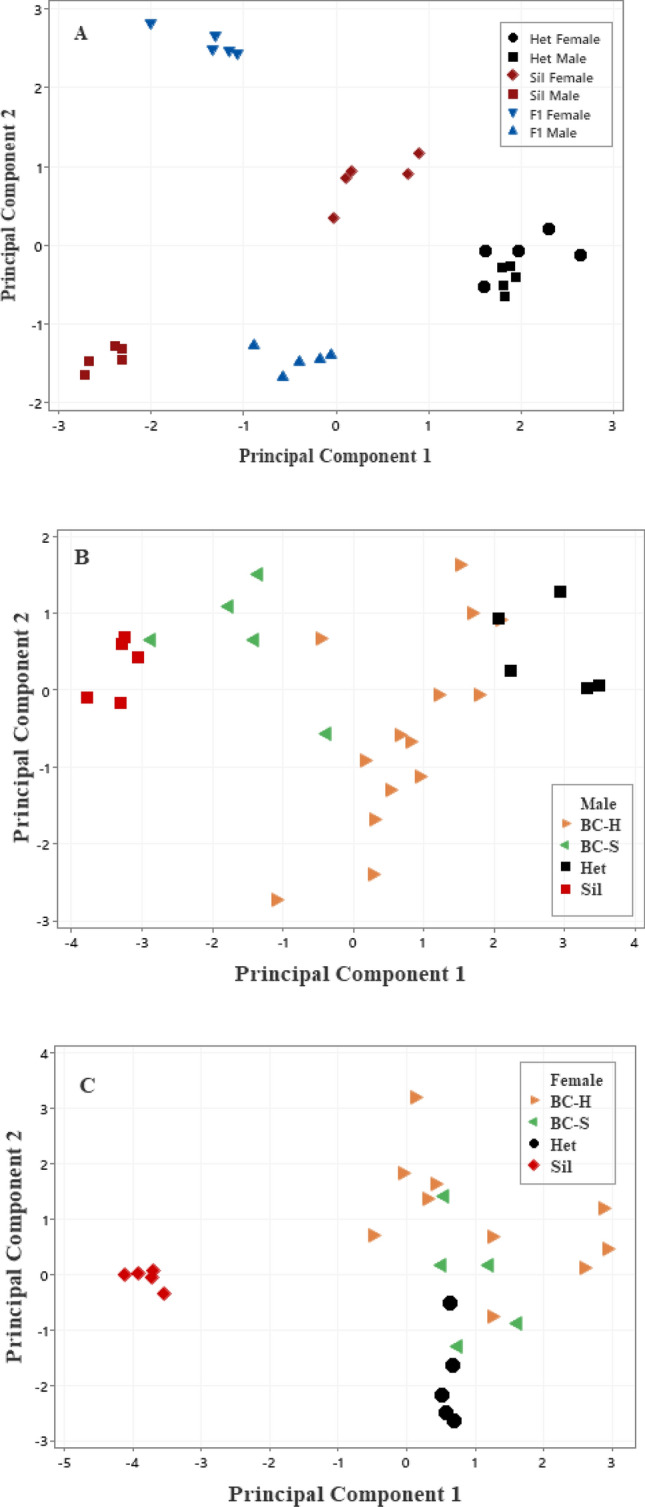
Scatterplot of principal component scores with ordinations representing differences in CHC compositions among genotypes for PC1 and PC2. (**A**) Parental and F1 hybrid females and males from the analysis of the six compounds found in F1 individuals. PC1 explained 47.2%, and PC2 explained 34.5% of the overall variation in the six CHCs (see Table S5). (**B**) Parental and backcross males from analysis of all nine compounds found in parental and backcross individuals. PC1 explained 51.6%, and PC2 explained 13.5% of the overall variation in the nine CHCs (see Table S9). (**C**) Parental and backcross females from analysis of all nine compounds found in parental and backcross individuals. PC1 explained 49.3%, and PC2 explained 21.6% of the overall variation in the nine CHCs (see Table S10. Symbols: *D. heteroneura* (Het), *D. silvestris* (Sil), F1 Hybrid (F1), BC—H backcross to *D. heteroneura,* and BC—S backcrossed to *D. silvestris*.

*Drosophila heteroneura* and *D. silvestris* males exhibited significant differences for both PC1 and PC2 scores with F1 males intermediate between the two parental males on PC1 and similar to *D. silvestris* on PC2 (Fig. 2A and Table S6). *D. heteroneura* and *D. silvestris* females were more similar but significantly different for PC1 and PC2 with F1 females outside the range and significantly different from females of the two parental species for both PC1 and PC2 (Table S6). Interestingly, the F1 females and males differed significantly for PC1 and PC2 scores. *D. silvestris*, but not *D. heteroneura*, showed strong and significant sexual dimorphism for both PC1 and PC2 scores (Fig. 2A and Table S4).

For all but one individual compound, the mean relative abundances of CHCs differed significantly between *D. heteroneura* and *D. silvestris* males (Table 1 and Table S3). Four of these compounds also differed in abundances between *D. heteroneura* and *D. silvestris* females: 2Me26, 2MeC30, and 11,15diM3C31 and 11,15diMeC33. *D. silvestris* females and males differed significantly in the relative abundances of five compounds (2MeC28, 2MeC30, 11,15diMeC31, 11 + 13MeC33, and 11,15diMeC33), while *D. heteroneura* females and males differed significantly for three compounds (2Me26, 11,15diMeC31, 2MeC32) (Table 1 and Table S3). For F1 males, the mean relative abundances of three compounds were intermediate between those for *D. heteroneura* and *D. silvestris* males (2Me26, 2Me30, 11 + 13MeC33), while the abundances of three compounds were less than those of both parental species. For F1 females, the mean abundances of two compounds were close to those of *D. silvestris* females (2Me26 and 2Me30), while three compounds were less abundant, and one compound was more abundant, in F1 females compared to females of the two parental species.

### Parental and backcross analyses

All nine CHCs that were detected in the parental species were also detected in backcross males and females (Tables 2 and 3) with the abundances of some of the CHCs significantly correlated within each sex (Tables S7 and S8). The PCA resulted in PC1 explaining 51.6% of the total variation for male genotypes and 49.3% for female genotypes (Tables S9 and S10). There were similar positive loadings for six of the CHCs and negative loadings for two of the CHCs for both males and females. PC2 explained 13.5% and 21.6% of the total variation in CHC composition of males and females, respectively. The three remaining principal components explained 10% or less of the total variation for both males and females (Tables S9 and S10).

**Table 2:** Variance (ANOVA) in the major CHCs of *D. heteroneura* (n = 5), *D. silvestris* (n = 5), and backcross males: BC—H backcross to *D. heteroneura* (n = 14), and BC—S backcross to *D. silvestris* (n = 5).

Male Genotype	Compounds								
	2MeC26	2MeC28	2MeC30	11 + 13MeC31	11,15diMeC31	2MeC32	11 + 13MeC33	11,15diMeC33	11,15diMeC35
*D. heteroneura*	11.19 (1.04) ^A^	19.61 (1.74) ^A^	7.44 (1.06) ^C^	5.72 (1.44) ^A^	13.19 (1.71) ^A^	5.20 (0.87) ^A^	6.37(1.32) ^A^	24.36(2.41) ^B^	6.92(0.89) ^A^
BC—H	8.99 (3.32) ^A^	19.28 (3.73) ^A^	16.07 (4.29) ^B^	4.84 (1.37) ^A^	6.81 (3.15) ^B^	5.23 (2.64) ^A^	5.12 (0.89) ^AB^	28.14 (3.24) ^AB^	5.52 (2.56) ^A^
BC—S	4.72 (1.26) ^B^	9.26 (3.71)^B^	33.91 (7.85) ^A^	2.93 (1.02) ^B^	6.54 (2.16) ^B^	3.92 (0.87)^A^	5.25 (0.93) ^AB^	28.81 (2.72) ^AB^	4.65 (1.28) ^A^
*D. silvestris*	2.03 (0.76) ^B^	8.85 (0.65)^B^	41.12 (4.13) ^A^	1.61 (0.23) ^B^	3.97 (0.449) ^B^	2.85 (0.67) ^A^	3.97 (0.43) ^B^	31.70 (3.34) ^A^	3.91 (0.84) ^A^
	F = 15.29	F = 23.25	F = 61.02	F = 13.12	F = 12.21	F = 2.12	F = 5.61	F = 4.86	F = 2.18
	df = 3, 25	df = 3, 25	df = 3, 25	df = 3, 25	df = 3, 25	df = 3, 25	df = 3,25	df = 3,25	df = 3,25
	P < 0.001	P < 0.001	P < 0.001	P < 0.001	P < 0.001	P = 0.123	P = 0.004	P = 0.008	P = 0.116

The mean percentage of each compound for each group of males with the standard deviation in parentheses is reported. Means that do not share a letter are significantly different following Tukey’s multiple comparison tests (P < 0.05)).

**Table 3:** Analysis of variance (ANOVA) of the major CHCs of *D. heteroneura* (n = 5), *D. silvestris* (n = 5), and backcross females: BC—H backcross to *D. heteroneura* (n = 10, and BC—S backcross to *D. silvestris* (n = 5).

Female Genotype	Compounds								
	2MeC26	2MeC28	2MeC30	11 + 13MeC31	11,15diMeC31	2MeC32	11 + 13MeC33	11,15diMeC33	11,15diMeC35
*D. heteroneura*	7.63 (1.94) ^AB^	20.23 (1.69)^B^	7.083 (0.378)^C^	6.23(0.95) ^AB^	12.14 (0.90)^A^	4.27 (0.91)^A^	5.44 (1.22)^A^	28.59 (1.37)^A^	8.39 (0.66)^A^
BC—H	9.35 (3.44) ^A^	33.40 (4.33)^A^	14.73 (4.60)^B^	5.39 (2.16) ^B^	12.76 (5.12)^A^	4.38 (1.96)^A^	3.51 (1.04)^B^	12.43 (10.79)^B^	4.06 (2.22)^B^
BC—S	4.64 (0.78) ^BC^	26.42 (6.24)^B^	18.42(3.46)^B^	8.43 (2.41) ^A^	14.40 (3.16)^A^	4.60 (2.25)^A^	4.33 (1.41)^AB^	15.09 (4.05)^B^	3.65 (1.74)^B^
*D. silvestris*	1.83 (0.32)^C^	12.28 (1.01)^C^	31.74 (0.59)^A^	2.00 (0.33) ^C^	1.13 (0.24)^B^	1.87 (0.25)^A^	2.50 (0.32)^B^	41.15 (1.82)^A^	5.50 (0.22)^AB^
	F = 12.04	F = 33.71	F = 75.70	F = 10.786	F = 14.49	F = 3.11	F = 6.98	F = 19.78	F = 9.20
	df = 3, 21	df = 3, 21	df = 3, 21	df = 3, 21	df = 3, 21	df = 3, 21	df = 3,21	df = 3,21	df = 3,21
	P < 0.001	P < 0.001	P < 0.001	P < 0.001	P < 0.001	P = 0.048	P = 0.002	P < 0.001	P < 0.001

The mean percentage of each compound for each group of females with the standard deviation in parentheses is reported. Means that do not share a letter are significantly different following Tukey’s multiple comparison tests (P < 0.05).

PC1 scores for CHC abundance in the two classes of backcross males were closer to the parental species to which they were backcrossed but unique to each class of males (Fig. 2B,C; Table S11). For PC2 and PC3, the backcross males were not significantly different from *D. silvestris* and *D. heteroneura* males. Both types of backcross females were closer in overall CHC abundances to *D. heteroneura* females for PC1 and significantly different from *D. silvestris;* in contrast, for PC2, both backcross females were significantly different from *D. heteroneura* but not from *D. silvestris* females (Fig. 2C, Table S12).

The abundances of individual compounds showed a range of patterns across backcross and parental-species genotypes. Four compounds (2MeC26, 2MeC28, 2MeC30, and 11 + 13MeC31) differed significantly in abundance between BC-H and BC-S backcross males, being similar in abundances to the same compounds in the parental species to which they were backcrossed. For the 11,15diMeC31 compound, the backcross males were significantly different from *D. heteroneura* and similar to *D. silvestris* (Table 2). The other compounds (2MeC32, 11 + 13MeC33, 11,15diMeC33, 11,15diMeC35) did not differ between the BC-H and BC-S backcross males, generally showing abundances intermediate to those of males of the two parental species. Similarly, the abundances of individual compounds in BC-H and BC-S backcross females differed significantly from each other for three compounds (Table 3: 2MeC26, 2MeC28, and 11 + 13MeC31). For 2MeC30 and 11,15diMeC33, the two types of backcross females were similar to each other and significantly different from both parental species with the abundance of 11,15diMeC33 outside the range of the parental species. The two backcross females differed significantly from *D. silvestris*, but not *D. heteroneura* for 11,15diMeC31, and both backcross females differed from *D. heteroneura* but not *D. silvestris* for 11,15diMeC35. The abundance of 2MeC32 and 11,15MeC33 in the backcross females did not differ significantly from that for *D. silvestris,* with BC-H females showing significant differences from *D. heteroneura* for 11,15MeC33 (Table 3).

## Discussion

This study examined the abundances of nine CHC compounds in two sympatric Hawaiian *Drosophila* species and their hybrids and found significant differences between the species and evidence of phenotypic disruption in both F1 and backcross hybrids. The differences in the correlation structure of CHC abundances between *D. heteroneura* and *D. silvestris* suggests that there may be an alteration in the regulation of CHC production that contributes to the phenotypic disruption in the hybrids. The species also differed in the abundances of most of the nine CHCs measured, with *D. silvestris* males exhibiting unique patterns of the overall abundances and ratios of compounds expressed. Alves et al.^13^ also observed that *D. silvestris* males exhibited the greatest differences in CHC abundances compared to two other closely related species in the *planitibia* subgroup, *D. hemipeza* from Oahu and *D. planitibia* from Maui. This suggests that there may have been a recent evolutionary change in CHC production in males of *D. silvestris* during the relatively brief history of this young species on Hawaii Island.

Phenotypic disruption of CHCs was extensive in both female and male hybrids between *D. silvestris* and *D. heteroneura*. Three of the nine CHCs were absent in F1 hybrids of both sexes, which translated to overall lower absolute CHC production in F1 hybrids compared to the parental species. For the six other compounds, F1 individuals were intermediate for PC1 scores but outside the range of the two parental species for PC2 scores with some individual compounds intermediate and others outside of the range of the parental species. Interestingly, all nine compounds were detected in backcross hybrids, where they showed greater variation in abundances in both females and males with some individuals more like the parental species and others more similar to the F1 hybrids. This type of alteration in F1 and backcrossed hybrids has also been shown in *D. simulans* and *D. sechellia*^5,31^.

The presence of CHCs in F1 and backcross hybrids in abundances outside of the range observed in *D. silvestris* and *D. heteroneura*, including the complete absence of CHCs in F1 hybrids, suggests a disruption in the biochemical and regulatory processes underlying these compounds in hybrids as a result of divergence of the parental species. Inter-species hybrids often experience failures in gene expression and regulation, which, may contribute to phenotypic dysfunction^3^. Several types of gene interactions may underlie hybrid dysfunctions such as *cis*–*trans* regulation and post-transcriptional processes, including mRNA splicing and processing^4,5^. CHC biosynthesis involves long-chain fatty acid synthesis via elongation, the transformation of long-chain fatty acids to aldehydes, and an oxidative decarboxylation phase^9,11,20,32^. The suppression or disruption of any gene involved in the biosynthesis of CHCs may lead to the loss or alteration of an enzyme necessary to produce a critical precursor essential for proper CHC synthesis^32,33^. For example, the oenocyte-specific knockdown in *D. melanogaster* of the expression of Cyp4g1, a gene involved in transforming aldehydes to hydrocarbons, resulted in a significant loss of detectable CHCs^20^. It has also been shown that the disruption of the NADH dehydrogenases *CG8680* and *CG5599* results in increased CHC production in *D. melanogaster* females and males^21^. A particular elongase or enzyme involved in the production of the dimethyl C31 and methyl C32 components may have been disrupted during the formation of the F1 hybrids in this study, resulting in the missing compounds (11 + 13MeC31, 11,15diMeC31, and 2MeC32). Desaturases and elongases involved in CHC production are known to evolve rapidly may contribute to between-sex variation, speciation and phenotypic disruption in hybrids^34–36^.

Evolutionary changes in the *cis*-regulatory regions of genes in the biochemical pathways of CHCs could lead to important differences in CHC abundances between closely related species^5,10,11,32^. For example, in *D. simulans* and *D. mauritiana,* hybrid females display CHC profiles that are intermediate to, but significantly different from, the two parental species, consistent with divergence at *cis*-regulatory regions^36^. Throughout the *Drosophila* genus the expression of the desaturase, DESAT-F, is correlated with long-chain CHC production, and this compound has undergone numerous alterations^37^. Due to the specificity of these pathways, it is possible that in closely related species there has been a change in the regulation of genes involved in the production of some CHCs^11^. CHC production may also involve complex interactions between genes on different chromosomes that result in altered phenotypes in hybrids^11^. For example, studies conducted by Noor and Coyne^38^ correlated two CHCs in *D. pseudoobscura* and *D. persimilis* with X and second chromosome effects in backcross males. However, in backcross females only the second chromosome significantly influenced the CHC phenotype. There is the potential for epistatic genetic effects in the mating isolation between *D. silvestris* and *D. heteroneura*^28^, and the difference in head shape in *D. silvestris* and *D. heteroneura* has been shown to have an X-effect and some autosomal genetic effects^26,39^.

The results presented here add to a growing number of studies that demonstrate that hybrids between species can experience substantial changes in gene expression and regulation contributing to phenotypic disruption^1–3,5^. In genus *Drosophila*, hybrid male sterility has been associated with changes in gene expression in F1 hybrids of *D. simulans* and *D. mauritiana*^40,41^, *D. melanogaster* and *D. simulans*^42^, *D. pseudoobscura pseudoobscura* and *D. p. bogotana*^43,44^ and F1 and backcross hybrids in two Hawaiian picture-wing *Drosophila*, *D. planitibia* and *D. silvestris*^45,46^. Similarly, hybrid disruption for brain morphology and neural gene expression was recently shown in two closely related sympatric *Heliconius* butterfly species^1^.

The differences in the relative abundances and ratios of CHC compounds between *D. silvestris* and *D. heteroneura* reported here and by Alves et al.^13^ suggests that CHCs may contribute to the behavioral reproductive isolation between these species^27,47,48^. Evolutionary changes in chemosensory systems between species have been shown to contribute to reproductive isolation and speciation through changes in the production and reception of CHCs^49^. These changes can involve the gain or loss of specific compounds^49^ or changes in the ratios of compounds^5,32,50^. Furthermore, the phenotypic disruption of CHCs could decrease F1 and backcross hybrid fitness through reduced dessication resistance and mating with parental species.

In summary, the two Hawaiian picture-wing *Drosophila*, *D. heteroneura* and *D. silvestris*, differed in the abundances of several CHCs and showed sexual dimorphism for some of these compounds. *D. silvestris* males appear to have diverged to a greater extent in CHC abundances compared to males of other species within the *planitibia* clade of Hawaiian picture-wing *Drosophila*^13^. The phenotypic disruption in F1 and backcross hybrids may have important consequences for the survival or reproductive success of hybrid individuals. These results also suggest that the biochemical pathways underlying CHC synthesis have diverged between these two closely related species. Additional studies are required with more extensive sampling, additional genetic analyses (e.g., Quantitative Trait Loci analyses) combined with genomic and gene expression analyses to better understand the changes in CHC production and the associated biochemical pathways^43,44,51,52^. Although the function of these CHCs in *D. silvestris* and *D. heteroneura* are still unknown, divergence in CHC abundance has been recent, as the two species appear to have diverged less than 1 million years ago^23^. It will be important to determine whether the differences in CHC abundances and the significant sequence divergence for chemosensory genes^30^ observed in these species has resulted in changes in chemosensory responses in *D. silvestris* and *D. heteroneura* and contribute to the behavioral reproductive isolation between them^27,48^.

## Supplementary Information

Supplementary Information.
