# Supplementary material for: Phenotypic disruption of cuticular hydrocarbon production in hybrids between sympatric species of Hawaiian picture-wing Drosophila
Source: Sci Rep. 2022 Mar 22;12:4865. doi: 10.1038/s41598-022-08635-w (PMC8941103; doi:10.1038/s41598-022-08635-w)
Supplement: Supplementary file 1 — Supplementary Information. [file 41598_2022_8635_MOESM1_ESM.docx]

**Supplemental Tables**

**Table S1.** Pearson’s correlation coefficients and associated P-values for the nine CHCs detected in *D. heteroneura* males (n = 10; above the diagonal) and *D. silvestris* males (n = 10; below the diagonal).

|  | Compounds | | | | | | | |  |
| --- | --- | --- | --- | --- | --- | --- | --- | --- | --- |
|  | 2MeC26 | 2MeC28 | 2MeC30 | 11 + 13MeC31 | 11,15diMeC31 | 2MeC32 | 11 + 13MeC33 | 11,15diMeC33 | 11,15diMeC35 |
| 2MeC26 |  | -0.548 -0.561 0.507 **0.702** -0.040 **0.919** **-0.913**  P=0.101 P=0.091 P=0.134 **P=0.024** P=0.914 **P<0.001** **P<0.001** | | | | | | | -0.130  P=0.721 |
| 2MeC28 | -0.232  P=0.518 | 0.303 -0.352 -0.581 -0.552 -0.559 0.590  P=0.394 P=0.319 P=0.078 P=0.098 P=0.093 P=0.072 | | | | | | | -0.234  P=0.515 |
| 2MeC30 | 0.112  P=0.758 | -0.563  P=0.090 | 0.336 **-0.934** -0.057 -0.275 0.463  P=0.342 **P<0.001** P=0.875 P=0.443 P=0.178 | | | | | | -0.265  P=0.460 |
| 11 + 13MeC31 | -0.108  P=0.767 | **0.737**  **P=0.015** | -0.252  P=0.482 | -0.107 -0.072 **0.764** -0.623  P=0.768 P=0.843 **P=0.010** P=0.055 | | | | | -0.135 P=0.711 |
| 11,15diMeC31 | 0.181  P=0.617 | -0.573  P=0.084 | 0.384  P=0.273 | -0.533  P=0.113 | 0.176 0.465 **-0.647**  P=0.626 P=0.175 **P=0.043** | | | | 0.314  P=0.377 |
| 2MeC32 | 0.051  P=0.889 | -0.515  P=0.127 | 0.233  P=0.517 | -0.444  P=0.198 | 0.617  P=0.057 | 0.014 -0.164  P=0.969 P=0.651 | | | 0.495  P=0.146 |
| 11 + 13MeC33 | 0.216  P=0.549 | **-0.806**  **P=0.005** | 0.626  P=0.053 | -0.507  P=0.135 | **0.680**  **P=0.030** | 0.341  P=0.335 | **-0.940**  **P<0.001** | | -0.043  P=0.907 |
| 11,15diMeC33 | 0.227  P=0.528 | **-0.962**  **P<0.001** | 0.435  P=0.209 | -0.630  P=0.057 | 0.468  P=0.173 | 0.477  P=0.163 | **0.807**  **P=0.005** |  | -0.210  P=0.561 |
| 11,15diMeC35 | 0.260  P=0.469 | -0.513  P=0.129 | -0.108  P=0.766 | -0.125  P=0.731 | 0.399  P=0.253 | 0.482  P=0.159 | 0.337  P=0.341 | 0.625  P=0.053 |  |

**Table S2.** Pearson’s correlation coefficients and associated P-values for the nine CHCs detected in *D. heteroneura* females (n = 10; above the diagonal) and *D. silvestris* females (n = 10; below the diagonal).

|  | Compounds | | | | | | | |  |
| --- | --- | --- | --- | --- | --- | --- | --- | --- | --- |
|  | 2MeC26 | 2MeC28 | 2MeC30 | 11 + 13MeC31 | 11,15diMeC31 | 2MeC32 | 11 + 13MeC33 | 11,15diMeC33 | 11,15diMeC35 |
| 2MeC26 |  | 0.317 -0.279 -0.142 0.071 -0.195 -0.195 -0.137  P=0.373 P=0.435 P=0.695 P=0.845 P=0.589 P=0.589 P=0.705 | | | | | | | -0.316  P=0.373 |
| 2MeC28 | 0.619  P=0.056 | 0.296 **-0.735** -0.552 -0.399 -0.449 0.370  P=0.407 **P=0.016** P=0.122 P=0.254 P=0.193 P=0.293 | | | | | | | **-0.786**  **P=0.007** |
| 2MeC30 | -0.428  P=0.218 | **-0.937**  **P<0.001** | -0.310 -0.310 -0.179 0.147 0.091  P=0.384 P=0.384 P=0.621 P=0.685 P=0.803 | | | | | | -0.366  P=0.298 |
| 11 + 13MeC31 | **0.654**  **P=0.040** | **0.735**  **P=0.015** | **-0.672**  **P=0.033** | -0.190 0.159 0.115 -0.415  P=0.151 P=0.661 P=0.752 P=0.233 | | | | | 0.578 P=0.080 |
| 11,15diMeC31 | 0.464  P=0.176 | **0.700**  **P=0.024** | **-0.796**  **P=0.006** | 0.536  P=0.111 | **0.783** -0.173 **-0.965**  **P=0.007** P=0.632 **P<0.001** | | | | **0.780**  **P=0.008** |
| 2MeC32 | 0.474  P=0.166 | -0.587  P=0.075 | **-0.690**  **P=0.027** | **0.660**  **P=0.038** | **0.916**  **P<0.001** | -0.048 **-0.763**  P=0.895 **P=0.010** | | | **0.774**  **P=0.009** |
| 11 + 13MeC33 | 0.270  P=0.450 | **0.838**  **P=0.002** | **-0.957**  **P<0.001** | 0.593  P=0.071 | **0.753**  **P=0.012** | **0.634**  **P=0.049** | -0.199  P=0.581 | | 0.345  P=0.329 |
| 11,15diMeC33 | -0.612  P=0.060 | **-0.798**  **P=0.006** | **0.803**  **P=0.005** | **-0.763**  **P=0.010** | **-0.926**  **P<0.001** | **-0.874**  **P<0.001** | **-0.740**  **P=0.014** |  | **-0.729**  **P=0.017** |
| 11,15diMeC35 | -0.177  P=0.626 | 0.477  P=0.163 | **-0.643**  **P=0.045** | 0.175  P=0.629 | 0.616  P=0.058 | 0.392  P=0.263 | **0.677**  **P=0.031** | -0.548  P=0.101 |  |

**Table S3.** Analysis of Variance (ANOVA) of major CHCs (in nanograms) of male and female *D. heteroneura*, *D. silvestris*, and F1 hybrids (n = 30 total individuals, n = 5 for each genotype). The mean of each compound is reported for each genotype with the standard deviation in parentheses. For each compound, means that do not share a letter are significantly different following a Tukey’s multiple-comparison test (P < 0.05) . The total of all nine compounds are reported for including the F1 hybrids that were missing compounds 11 + 13MeC31, 11,15diMeC31, and 11,15diMeC31.

| Compounds | | | | | | | | | |  |
| --- | --- | --- | --- | --- | --- | --- | --- | --- | --- | --- |
| Genotypes | 2MeC26 | 2MeC28 | 2MeC30 | 11 + 13MeC31 | 11,15diMeC31 | 2MeC32 | 11 + 13MeC33 | 11,15diMeC33 | 11,15diMeC35 | Total  CHC |
| *D. heteroneura* Male | 182 (14.3)^A^ | 483 (41.0)^B^ | 215 (26.3)^CD^ | 111 (26.2)^A^ | 184 (30.3)^A^ | 109 (22.1)^A^ | 89 (15.6)^CD^ | 716 (71.7)^AB^ | 147 (20.8)^A^ | 2236 |
| *D. silvestris* Male | 38 (13.5)^C^ | 688 (82.8)^A^ | 800 (97.4)^A^ | 56 (17.5)^B^ | 73 (13.0)^C^ | 49 (10.9)^B^ | 55 (13.1)^D^ | 380 (50.8)^C^ | 74 (11.7)^B^ | 2213 |
| F1 Male | 125 (20.0)^B^ | 388 (39.4)^C^ | 249 (45.5)^C^ | -- | -- | -- | 82 (7.4)^CD^ | 255 (26.6)^D^ | 46 (18.2)^B^ | 1145 |
| *D. heteroneura*  Female | 150 (17.7)^B^ | 411(37.2)^BC^ | 132 (19.0)^D^ | 99 (27.6)^A^ | 137 (25.0)^B^ | 53 (10.0)^B^ | 100 (15.8)^BC^ | 672 (28.2)^B^ | 120 (20.5)^A^ | 1874 |
| *D. silvestris* Female | 54 (14.3)^C^ | 408 (36.4)^BC^ | 477 (64.4)^B^ | 72 (16.6)^AB^ | 67 (23.9)^C^ | 84 (28.8)^AB^ | 130 (27.2)^B^ | 798 (90.3)^A^ | 149 (32.7)^A^ | 2239 |
| F1 Female | 52 (16.6)^C^ | 138 (13.2)^D^ | 476 (38.2)^B^ | -- | -- | -- | 246 (26.4)^A^ | 438 (47.5)^C^ | 63 (13.7)^B^ | 984 |
|  | F= 68.84 | F= 72.54 | F= 98.87 | F= 10.48 | F= 27.29 | F= 10.48 | F= 64.07 | F= 65.91 | F= 23.32 |  |
|  | df= 5, 24 | df= 5, 24 | df= 5, 24 | df= 3, 16 | df= 3, 16 | df= 3, 16 | df= 5,24 | df= 5,24 | df= 5,24 |  |
|  | P<0.001 | P <0.001 | P <0.001 | P < 0.001 | P <0.001 | P < 0.001 | P <0.001 | P <0.001 | P <0.001 |  |

**Table S4**. Pearson’s correlation coefficients and associated P-values for the six CHCs detected in male and female *D. heteroneura*, *D. silvestris* and F1 Hybrids (n = 30 total individuals, n = 5 for each genotype).

|  | Compounds | | | | | |
| --- | --- | --- | --- | --- | --- | --- |
|  | 2MeC26 | 2MeC28 | 2MeC30 | 11 + 13MeC33 | 11,15diMeC33 | |
| 2MeC28 | 0.326  P=0.079 |  | | | | |
| 2MeC30 | **-0.774**  **P<0.001** | -0.096  P=0.613 |  | | | |
| 11 + 13MeC33 | -0.160  P=0.400 | **-0.821**  **P<0.001** | 0.223  P=0.235 |  |  |  |
| 11,15diMeC33 | 0.241  P=0.200 | -0.460  P=0.010 | **-0.746**  **P<0.001** | 0.071  P=0.710 |  |  |
| 11,15diMeC35 | 0.283  P=0.130 | -0.196  P=0.300 | **-0.708**  **P<0.001** | -0.167  P=0.377 | **0.763**  **P<0.001** |  |

**Table S5.** Principal component (PC) loadings and percent variation explained for six CHCs in *D. heteroneura*, *D. silvestris* and F1 Hybrids: (n = 30 total individuals, n = 5 for each genotype).

| Compound | PC1 | PC2 | PC3 | PC4 | PC5 | PC6 |
| --- | --- | --- | --- | --- | --- | --- |
| 2MeC26 | 0.392 | -0.263 | -0.698 | 0.142 | -0.474 | 0.212 |
| 2MeC28 | -0.025 | -0.689 | 0.016 | 0.015 | 0.553 | 0.468 |
| 2MeC30 | -0.576 | 0.089 | 0.179 | 0.198 | -0.451 | 0.622 |
| 11+13MeC33 | -0.120 | 0.588 | -0.517 | 0.200 | 0.514 | 0.263 |
| 11,15diMeC33 | 0.497 | 0.298 | 0.226 | -0.588 | -0.033 | 0.516 |
| 11,15diMeC35 | 0.503 | 0.118 | 0.403 | 0.745 | 0.036 | 0.122 |
| **Percent Variation Explained** | **47.2** | **34.5** | **13.9** | **2.5** | **0.09** | **0.01** |

**Table S6.** Analysis of Variance (ANOVA) of the principal component scores from the PCA of CHCs (in percent abundances) in male and female *D. heteroneura*, *D. silvestris*, and F1 hybrids (n = 30 total individuals, n = 5 for each genotype). The mean of each principal component score is reported for each genotype with the standard deviation in parentheses. Means that do not share a letter are significantly different following Tukey’s multiple comparison test (P < 0.05) .

|  | CHC Principal Components | | |
| --- | --- | --- | --- |
| Genotypes | PC 1 | PC 2 | PC 3 |
| *D. heteroneura* Male | 1.85 (0.06)^A^ | -0.42 (0.16) ^C^ | 0.03 (0.26) ^B^ |
| *D. silvestris* Male | -2.48 (0.20)^E^ | -1.43 (0.14)^D^ | 0.85 (0.25)^A^ |
| F1 Male | -0.42 (0.33)^C^ | -1.47 (0.15)^D^ | -1.33 (0.17)^D^ |
| *D. heteroneura* Female | 2.03 (0.45)^A^ | -0.12 (0.26)^C^ | -0.07 (0.15)^B^ |
| *D. silvestris* Female | 0.39 (0.42)^B^ | 0.84 (0.30)^B^ | 1.25 (0.30)^A^ |
| F1 Female | -1.37 (0.38)^D^ | 2.59 (0.18)^A^ | -072 (0.13)^C^ |
|  | F= 142.72 | F= 279.88 | F= 68.39 |
|  | df= 5, 24 | df= 5, 24 | df= 5, 24 |
|  | P <0.001 | P <0.001 | P <0.001 |

**Table S7.** Pearson’s correlation coefficients and associated P-values for the nine CHCs detected in males (*D. heteroneura* n = 5*, D. silvestris* n = 5*,* and backcross males*:* BC – H backcross to *D. heteroneura* n = 14 and BC – S backcross to *D. silvestris* n = 5).

|  | Compounds | | | | | | | |
| --- | --- | --- | --- | --- | --- | --- | --- | --- |
|  | 2MeC26 | 2MeC28 | 2MeC30 | 11 + 13MeC31 | 11,15diMeC31 | 2MeC32 | 11 + 13MeC33 | 11,15diMeC33 |
| 2MeC28 | **0.700**  **P<0.001** |  | | | | | | |
| 2MeC30 | **-0.796**  **P<0.001** | **-0.863**  **P<0.001** |  | | | | | |
| 11 + 13MeC31 | **0.513**  **P=0.004** | **0.689**  **P=0.001** | **-0.755**  **P<0.001** |  | | | | |
| 11,15diMeC31 | **0.458**  **P=0.012** | 0.206  P=0.283 | **-0.576**  **P<0.001** | **0.423**  **P=0.022** |  | | | |
| 2MeC32 | 0.182  P=0.343 | 0.203  P=0.291 | **-0.415**  **P=0.025** | **0.377**  **P=0.044** | 0.278  P=0.144 |  | | |
| 11 + 13MeC33 | 0.316  P=0.094 | 0.340  P=0.071 | **-0.479**  **P=0.009** | **0.604**  **P=0.001** | 0.348  P=0.065 | 0.130  P=0.502 |  | |
| 11,15diMeC33 | **-0.466**  **P=0.011** | -0.291  P=0.125 | **0.410**  **P=0.027** | **-0.576**  **P=0.001** | **-0.657**  **P<0.001** | -0.301  P=0.113 | **-0.374**  **P=0.045** |  |
| 11,15diMeC35 | 0.230  P=0.229 | 0.339  P=0.072 | **-0.578**  **P=0.001** | **0.406**  **P=0.029** | **0.607**  **P<0.001** | 0.257  P=0.178 | 0.225  P=0.240 | **-0.373**  **P=0.046** |

**Table S8.** Pearson’s correlation coefficients and associated P-values for the nine CHCs detected in female *D. heteroneura* n = 5, *D. silvestris* n = 5, and backcross individuals: BC – H backcross to *D. heteroneura* n= 10, and BC – S backcross to *D. silvestris* n = 5.

|  | Compounds | | | | | | | |
| --- | --- | --- | --- | --- | --- | --- | --- | --- |
|  | 2MeC26 | 2MeC28 | 2MeC30 | 11 + 13MeC31 | 11,15diMeC31 | 2MeC32 | 11 + 13MeC33 | 11,15diMeC33 |
| 2MeC28 | **0.622**  **P=0.001** |  | | | | | | |
| 2MeC30 | **-0.600**  **P=0.002** | **-0.489**  **P=0.013** |  | | | | | |
| 11 + 13MeC31 | 0.221  P=0.287 | 0.326  P=0.112 | **-0.436**  **P=0.029** |  | | | | |
| 11,15diMeC31 | **0.452**  **P=0.023** | **0.554**  **P=0.004** | **-0.650**  **<P0.001** | **0.646**  **P<0.001** |  | | | |
| 2MeC32 | 0.375  P=0.064 | 0.299  P=0.146 | **-0.504**  **P=0.010** | **0.563**  **P=0.003** | **0.647**  **P<0.001** |  | | |
| 11 + 13MeC33 | 0.148  P=0.479 | -0.084  P=0.691 | **0.450**  **P=0.024** | **-0.588**  **P=0.002** | **0.482**  **P=0.015** | **0.448**  **P=0.025** |  | |
| 11,15diMeC33 | **-0.579**  **P=0.002** | **-0.792**  **P<0.001** | 0.366  P=0.072 | **-0.646**  **P<0.001** | **-0.786**  **P<0.001** | **-0.567**  **P=0.003** | -0.263  P=0.205 |  |
| 11,15diMeC35 | -0.228  P=0.274 | **-0.453**  **P=0.023** | -0.176  P=0.401 | 0.125  P=0.553 | -0.014  P=0.947 | 0.025  P=0.905 | **0.415**  **P=0.039** | 0.243  P=0.242 |

**Table S9**. Principal component (PC) loadings and percent variation explained for nine CHCs for males (*D. heteroneura* n = 5, *D. silvestris* n =5, and backcross males: BC – H backcross to *D. heteroneura* n = 14 and BC – S backcross to *D. silvestris* n =5).

| Variable | PC1 | PC2 | PC3 | PC4 | PC5 | PC6 |
| --- | --- | --- | --- | --- | --- | --- |
| 2MeC26 | 0.352 | -0.308 | 0.002 | 0.283 | 0.472 | -0.334 |
| 2Mec28 | 0.353 | -0.506 | 0.161 | 0.122 | -0.077 | 0.260 |
| 2MeC30 | -0.435 | 0.203 | -0.186 | -0.123 | 0.116 | 0.203 |
| 11+13MeC31 | 0.393 | -0.123 | -0.106 | -0.313 | -0.122 | 0.443 |
| 11,15diMeC31 | 0.326 | 0.501 | -0.125 | 0.276 | 0.126 | -0.351 |
| 2MeC32 | 0.209 | 0.241 | 0.663 | -0.607 | 0.122 | -0.202 |
| 11+13MeC33 | 0.275 | -0.058 | -0.599 | -0.444 | -0.344 | -0.390 |
| 11,15diMeC33 | -0.318 | -0.368 | 0.272 | 0.073 | -0.493 | -0.490 |
| 11,15diMeC35 | 0.286 | 0.381 | 0.197 | 0.380 | -0.592 | 0.167 |
| **Percent Variation Explained** | **51.6** | **13.5** | **9.9** | **7.7** | **4.7** | **2.1** |

**Table S10**. Principal component (PC) loadings and percent variation explained for nine CHCs for females (*D. heteroneura* n = 5, *D. silvestris* n = 5, and backcross females: BC – H backcross to *D. heteroneura* n = 10 and BC – S backcross to *D. silvestris* n = 5).

| Variable | PC1 | PC2 | PC3 | PC4 | PC5 | PC6 |
| --- | --- | --- | --- | --- | --- | --- |
| 2MeC26 | 0.321 | 0.253 | 0.516 | 0.137 | 0.385 | -0.509 |
| 2Mec28 | 0.334 | 0.436 | 0.058 | -0.346 | -0.158 | 0.093 |
| 2MeC30 | -0.354 | 0.118 | -0.583 | 0.016 | 0.142 | -0.516 |
| 11+13MeC31 | 0.353 | -0.235 | -0.421 | -0.264 | 0.142 | 0.063 |
| 11,15diMeC31 | 0.424 | -0.057 | -0.108 | -0.076 | -0.219 | 0.251 |
| 2MeC32 | 0.353 | -0.146 | -0.155 | 0.765 | -0.382 | -0.161 |
| 11+13MeC33 | 0.245 | -0.508 | -0.030 | 0.051 | 0.642 | 0.092 |
| 11,15diMeC33 | -0.411 | -0.191 | 0.307 | 0.226 | 0.035 | 0.389 |
| 11,15diMeC35 | -0.033 | -0.598 | 0.286 | -0.383 | -0.424 | -0.462 |
| **Percent Variation Explained** | **49.3** | **21.6** | **9.8** | **5.7** | **4.7** | **4.0** |

**Table S11.** Analysis of Variance (ANOVA) for the principal component (PC) scores from the PCA of CHCs for males (*D. heteroneura* n = 5, *D. silvestris* n = 5, and backcross males: BC – H backcross to *D. heteroneura* n = 14 and BC – S backcross to *D. silvestris* n = 5. The mean of each principal component score is reported for each genotype with the standard deviation in parentheses. Means that do not share a letter are significantly different following Tukey’s multiple comparison tests (P < 0.05) .

|  | CHC Principal Components | | |
| --- | --- | --- | --- |
| Genotypes | PC 1 | PC 2 | PC 3 |
| *D. heteroneura* Male | 2.81 (0.28)^A^ | 0.503 (0.56)^A^ | -0.55 (1.11)^A^ |
| BC-H Male | 0.74 (0.89)^B^ | -0.52 (1.30)^A^ | 0.39 (0.98)^A^ |
| BC-S Male | -1.55 (0.90)^C^ | 0.67 (0.78)^A^ | -0.55 (0.63)^A^ |
| *D. silvestris* Male | -3.33 (0.26)^D^ | 0.29 (0.39)^A^ | -0.01 (0.38)^A^ |
|  | F= 61.67 | F= 2.45 | F= 2.22 |
|  | df=3, 25 | df= 3,25 | df= 3, 25 |
|  | P<0.001 | P=0.087 | P= 0.111 |

**Table S12.** Analysis of Variance (ANOVA) for the principal component scores from the PCA of CHCs for females (*D. heteroneura* n = 5, *D. silvestris* n = 5, and backcross females: BC – H backcross to *D. heteroneura* n = 10 and BC – S backcross to *D. silvestris* n = 5. The mean of each principal component score is reported for each genotype with the standard deviation in parentheses. Means that do not share a letter are significantly different following Tukey’s multiple comparison tests (P < 0.05) .

|  | CHC Principal Components | | |
| --- | --- | --- | --- |
| Genotypes | PC 1 | PC 2 | PC 3 |
| *D. heteroneura* Female | 0.62 (0.07)^A^ | -1.89 (0.86)^B^ | 1.13 (0.32)^A^ |
| BC-H Female | 1.12 (1.28)^A^ | 1.03 (1.08)^A^ | 0.21 (0.68)^B^ |
| BC-S Female | 0.93 (0.47)^A^ | -0.10 (1.06)^A^ | -1.26 (0.59)^C^ |
| *D. silvestris* Female | -3.79 (0.22)^B^ | -0.07 (0.17)^A^ | -0.29 (0.06)^B^ |
|  | F= 40.17 | F= 11.11 | F= 17.67 |
|  | df= 3, 21 | df= 3, 21 | df= 3, 21 |
|  | P <0.001 P <0.001 | P <0.001 | P <0.001 |
